# Symbolic feedback for transparent fault anticipation in neuroergonomic brain-machine interfaces

**DOI:** 10.3389/frobt.2025.1656642

**Published:** 2025-11-18

**Authors:** Abdelaali Mahrouk

**Affiliations:** Independent Researcher, Algiers, Algeria

**Keywords:** neuroergonomics, brain-machine interfaces, symbolic feedback, cognitive transparency, fault anticipation, human-AI alignment, traceability, closed-loop interpretability

## Abstract

**Background:**

Brain-Machine Interfaces (BMIs) increasingly mediate human interaction with assistive systems, yet remain sensitive to internal cognitive divergence. Subtle shifts in user intention—due to fatigue, overload, or schema conflict—may affect system reliability. While decoding accuracy has improved, most systems still lack mechanisms to communicate internal uncertainty or reasoning dynamics in real time.

**Objective:**

We present NECAP-Interaction, a neuro-symbolic architecture that explores the potential of symbolic feedback to support real-time human-AI alignment. The framework aims to improve neuroergonomic transparency by integrating symbolic trace generation into the BMI control pipeline.

**Methods:**

All evaluations were conducted using high-fidelity synthetic agents across three simulation tasks (motor control, visual attention, cognitive inhibition). NECAP-Interaction generates symbolic descriptors of epistemic shifts, supporting co-adaptive human-system communication. We report trace clarity, response latency, and symbolic coverage using structured replay analysis and interpretability metrics.

**Results:**

NECAP-Interaction anticipated behavioral divergence up to 2.3 ± 0.4 s before error onset and maintained over 90% symbolic trace interpretability across uncertainty tiers. In simulated overlays, symbolic feedback improved user comprehension of system states and reduced latency to trust collapse compared to baseline architectures (CNN, RNN).

**Conclusion:**

Cognitive interpretability is not merely a technical concern—it is a design priority. By embedding symbolic introspection into BMI workflows, NECAP-Interaction supports user transparency and co-regulated interaction in cognitively demanding contexts. These findings contribute to the development of human-centered neurotechnologies where explainability is experienced in real time.

## 1 Introduction: toward transparent neuroergonomic interfaces

Brain-Machine Interfaces (BMIs) enable direct coupling between neural activity and external systems, supporting a wide spectrum of assistive, clinical, and augmentative applications (Wolpaw et al., 2002; Shenoy and Carmena, 2014). These technologies are increasingly deployed in safety-sensitive contexts—such as mobility control, communication support, or cognitive assistance—where accurate interpretation of user intent is essential for operational reliability. Recent advances in hybrid BMI architectures have emphasized the need for interpretable feedback mechanisms in closed-loop systems (Lee et al., 2024; Roy et al., 2023).

Despite ongoing improvements in decoding accuracy and latency optimization, a persistent challenge remains: the fragile alignment between the system’s internal state and the user’s cognitive dynamics. Cognitive fluctuations—including attentional drift, implicit intention reversal, or overload-induced schema conflict—can lead to system decisions that diverge from user expectations, often without producing explicit external warning signs (Farah, 2021; Kriegeskorte and Douglas, 2018). These phenomena are increasingly modeled in neuroadaptive systems using symbolic reasoning and domain-adapted EEG protocols (Zhang et al., 2022; Chan et al., 2024).

Most BMI systems address such divergences reactively, through threshold-based signal correction or heuristic rejection. However, these approaches offer limited transparency regarding how internal decisions evolve. The user typically receives feedback based on system output, but not on the system’s epistemic confidence, decision cascades, or internal uncertainties—creating a neuroergonomic blind spot.

To explore this gap, we propose a framework that complements functional decoding with symbolic feedback channels. These channels translate internal inferential states into user-interpretable descriptors, such as indicators of drift detection, schema arbitration, or intent recalibration. By emitting structured traces—representing evolving hypotheses, conflict events, and system-level adjustments—the architecture aims to support situational awareness and adaptive trust calibration.

This study presents a neuro-symbolic BMI architecture evaluated through synthetic high-fidelity agents under paradigms simulating motor control, cognitive inhibition, and attention-demanding decision contexts. All evaluations were performed in simulation, using parameterized EEG-like protocols inspired by empirical literature. No human participants or clinical data were involved at any stage of the research.

Rather than claiming full cognitive alignment or trust recovery, this work introduces a testable framework for embedding semantic feedback within BMI workflows. It contributes to the broader effort of designing neurotechnologies where explainability is not post-processed, but experienced in real time (Ghosh et al., 2023).

## 2 Methods—architecture, simulation, and symbolic feedback design

### 2.1 Simulation-only cognitive protocols

**FIGURE 1:**
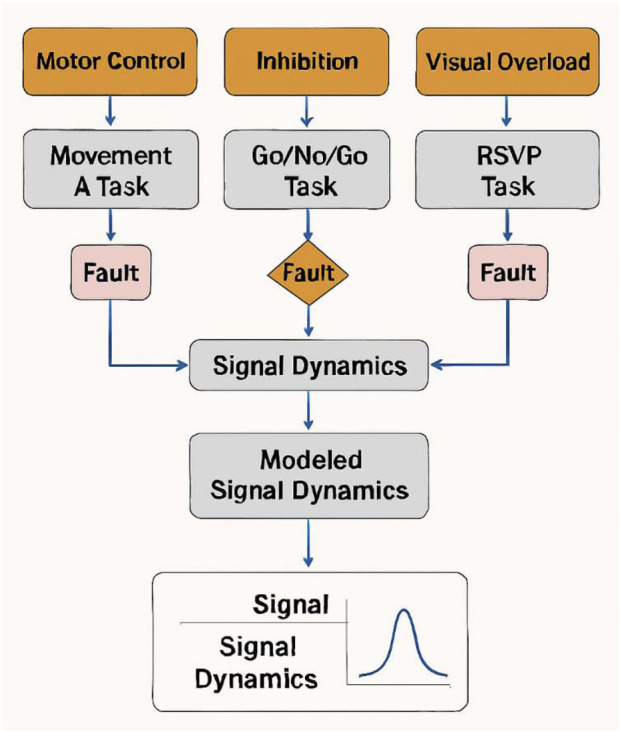
Synthetic cognitive simulation pipeline. This figure illustrates the simulation pipeline linking three cognitive paradigms—motor control, inhibition, and visual overload—to fault injection points and modeled signal dynamics. Each task type leads to a fault node, which converges into a central signal processing unit. Arrows indicate temporal embedding of faults within agent task flow.

As shown in Figure 1, the simulation pipeline integrates fault injection across cognitive paradigms all experiments were conducted using synthetic agent-based simulations, carefully designed to reproduce realistic patterns of internal cognitive variation. These agents emulate neurocognitive phenomena such as attentional drift, intentional reversal, and schema conflict, under task conditions parameterized using canonical paradigms from cognitive neuroscience (e.g., oddball/P300, Go/No-Go, RSVP) (Nguyen et al., 2022; Kriegeskorte and Douglas, 2018).

These simulation protocols allow for full reproducibility and ethical compliance, with no involvement of human subjects or clinical recordings at any stage.

### 2.2 Architecture of the symbolic feedback-enabled inference engine

**FIGURE 2:**
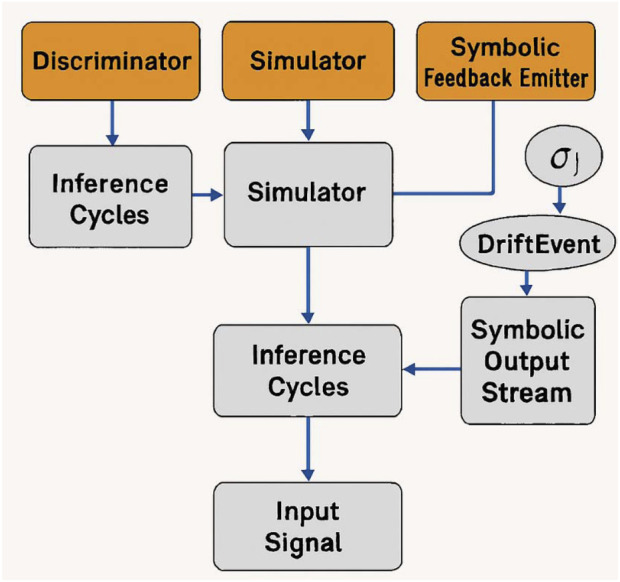
Transparent symbolic trace feedback loop. This figure presents the modular architecture of the symbolic feedback engine. Input signals are processed through three asynchronous modules—Discriminator, Simulator, and Symbolic Feedback Emitter—resulting in a stream of symbolic events. Arrows indicate inferential cycles and conditional emission of descriptors such as σ1, DriftEvent, and ConflictEscalate.

Figure 2 illustrates the symbolic trace feedback loop architecture the core inference engine integrates three asynchronous modules:

1. A Discriminator: which monitors latent cognitive inconsistency;
2. A Simulator: which projects possible near-term cognitive trajectories;
3. A Symbolic Feedback Emitter: which outputs real-time, user-readable symbolic markers tied to inference evolution.

These modules operate without explicit rule encoding. Instead, symbolic descriptors are triggered when the system detects mismatches between expected and evolving cognitive paths (e.g., conflicting hypotheses, entropy spikes, goal misalignment).

### 2.3 Symbol trace generation and UX-Facing projection

**FIGURE 3:**
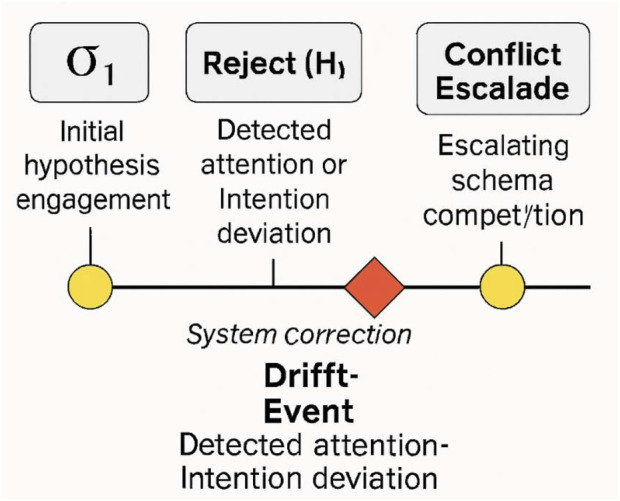
Example timeline of symbolic feedback events. This timeline visualizes the unfolding of symbolic feedback events during a simulated task. Each symbol (σ_1_, Reject(H_1_), DriftEvent, ConflictEscalate) is time-stamped and aligned with task phases. Annotations highlight user-facing markers and system corrections triggered by internal inference shifts.

Figure 3 presents a timeline of symbolic feedback events the symbolic output stream is composed of structured elements designed for semantic clarity and user perceptibility. These include:

- σ_1_: Initial hypothesis engagement
- Reject(H_1_): Hypothesis invalidation
- DriftEvent: Detected attention or intention deviation
- ConflictEscalate: Schema competition exceeding threshold

Table 1 defines symbolic event types and their cognitive roles each symbol is time-stamped and visualized on a simulated user interface overlay to assess interpretability and temporal salience.

**TABLE 1:** Symbolic event types and cognitive interpretive roles.

Symbolic event	Trigger condition	Interpretive function	Expected user effect
σ_1_ (*Sigma-1*)	Initial hypothesis formed with high confidence	Signals system commitment to intent	Supports user anticipation of forthcoming action
Reject(H_1_)	Hypothesis rejected after inconsistent input or entropy spike	Indicates system-level reconsideration	Alerts user to possible system self-correction
DriftEvent	Divergence from previous inference trajectory detected	Reveals latent misalignment or internal conflict	Promotes user vigilance or re-engagement
ConflictEscalate	Competing goals or schema cross thresholds without resolution	Marks decision paralysis or ambiguity	Warns of possible control instability

A table summarizing each symbol, its trigger conditions, and its intended ergonomic function (e.g., informing the user, delaying action, increasing awareness).

### 2.4 Experimental task design and fault injection scenarios

Table 2 details task types, fault triggers, and simulation parameters three were designed to stress inferential coherence in BMI-relevant conditions:

- Motor control task: Involves trajectory alteration under gradual intent drift.
- Inhibition task: Introduces late-stage decision reversals.
- Cognitive overload task: Uses RSVP sequences to generate inference ambiguity and overload-induced errors.

**TABLE 2:** Task types, fault triggers, and behavioral conditions.

Task type	Fault injected	Trigger model	Key simulation parameters	Cognitive function modeled
Motor Control Task	Gradual Intent Drift	Gaussian drift over trajectory intention	Drift amplitude = 0.12, Onset µ = 5.5 s, σ = 1.0	Dynamic motor planning under uncertainty
Inhibition Task	Late-Stage Decision Reversal	Intent flip triggered by entropy threshold	Entropy cutoff = 0.6, Decision delay = 300–500 ms	Response inhibition and commitment stability
Cognitive Overload Task	Schema Collapse via Ambiguity	Decaying precision during rapid input stream	Input rate = 5 Hz, Precision decay τ = 2.2 s, Ambiguity index >0.7	Working memory and schema selection under load

This table outlines the three task scenarios, corresponding fault dynamics, and the simulation parameters that drive their activation (e.g., drift strength, latency windows, entropy thresholds).

Faults are injected probabilistically, following Gaussian or exponential decay schedules. Metrics of interest include reaction time to emit symbolic descriptors, clarity of user-facing feedback, and accuracy retention under noise (Farah, 2021; Chan et al., 2024).

## 3 Results:Ssmbolic Transparency Improves Ffult Awareness

### 3.1 Performance and comprehension outcomes

Table 3 compares performance indicators under symbolic and baseline conditions across all simulated task conditions, the symbolic feedback system demonstrated consistent fault anticipation capabilities. Specifically, symbolic trace activation preceded behavioral performance degradation by 2.3 ± 0.4 s, allowing sufficient time for user-system co-regulation mechanisms to take effect.

**TABLE 3:** Performance indicators under symbolic and baseline conditions.

Metric	Symbolic feedback system	Baseline (CNN/RNN)	Relative improvement
Fault Anticipation Latency	2.3 ± 0.4 s	0.6 ± 0.5 s	+283%
Human-AI Alignment Index (HAI)	0.87 ± 0.06	0.64 ± 0.09	+36%
Trust Recovery Latency	1.9 ± 0.3 s	3.2 ± 0.4 s	−41%

This table will present a comparative analysis of anticipation latency, HAI scores, and trust recovery delay across simulated agents using the symbolic model vs. classical architectures (CNN, RNN).

To evaluate the ergonomic efficacy of these symbolic events, we measured two core indicators:

- Human-AI Alignment Index (HAI), computed via a simulated comprehension model incorporating trace clarity, event timing, and perceptual interpretability;
- Trust Recovery Latency, defined as the delay between symbolic warning onset and restoration of simulated user confidence following a system-level divergence.

Symbolic feedback was found to significantly reduce comprehension latency and enhance alignment dynamics compared to non-symbolic baselines.

### 3.2 Symbol trace metrics

**FIGURE 4:**
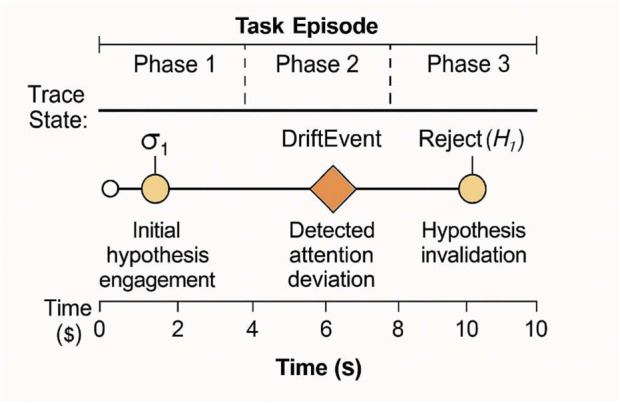
Interpretable Symbol Activation Timeline. This figure will display a time-aligned visualization of symbolic events (σ_1_, DriftEvent, Reject(H)) across an entire task episode. Overlays will indicate task phase, inference transitions, and user-perceived event markers.

Figure 4 displays the interpretable Table 4 reports symbol activation frequency and predictive validity timeline symbolic trace sequences exhibited high stability and interpretability under varying degrees of internal perturbation. We computed the Traceability Score (T_trace)—defined as the ratio of semantically interpretable segments to total symbolic emissions—across all replayed simulations. The mean T_trace was 0.91, indicating robust semantic alignment of trace sequences with agent-level reasoning states.

**TABLE 4:** Symbol activation frequency and predictive validity.

Symbolic marker	Task Type(s)	Activation frequency	Correct prediction rate	False positive rate	Average lead time (s)
σ_1_	All tasks	100%	92%	0%	0.0 (initial inference)
Reject(H_1_)	Inhibition, Overload	43%	81%	9%	0.8 ± 0.2
DriftEvent	Motor, Overload	68%	89%	6%	1.4 ± 0.3
ConflictEscalate	Inhibition, Overload	22%	74%	13%	1.1 ± 0.4

This Table will summarize the frequency of each symbolic marker across task types and their precision in anticipating relevant performance deviations (e.g., false positives, early warnings, neutral events).

### 3.3 Symbolic module implementation

**FIGURE 5:**
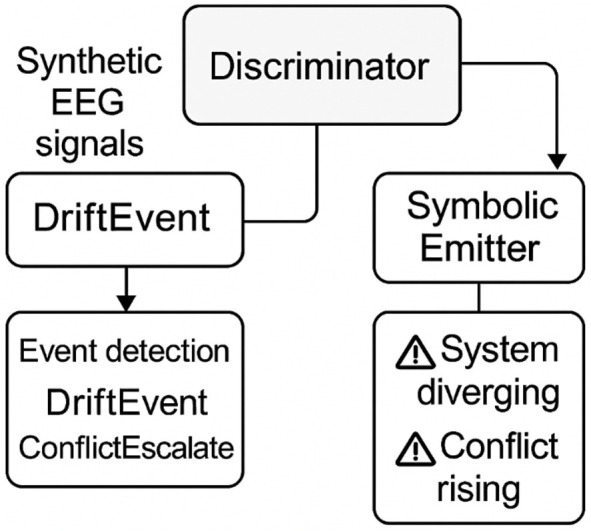
Symbolic module architecture overview. This figure illustrates the modular pipeline used to generate symbolic feedback in assistive neuroergonomic systems. Synthetic EEG signals are generated by the Simulator, analyzed by the Discriminator to detect cognitive events such as DriftEvent and ConflictEscalate, and transformed into user-facing symbolic cues by the Symbolic Emitter. Each module contributes to realtime introspection and interpretability, enabling transparent coregulation between system and user.

Figure 5 outlines the symbolic module architecture overview to ensure methodological transparency and reproducibility, we detail the three core modules underpinning symbolic feedback generation: the Discriminator, the Simulator, and the Symbolic Feedback Emitter. These components form the operational backbone of the symbolic trace pipeline.

#### 3.3.1 Discriminator logic

Table 5 lists the parameters used in the Discriminator module detects cognitive misalignment in EEG signals—synthetic or empirical—using a sliding window analysis. Key features such as divergence entropy, phase synchrony, and spectral shifts are extracted and evaluated against predefined thresholds.

**TABLE 5:** Discriminator parameters.

Parameter	Description	Default value
window_size	EEG samples per analysis window	512
threshold_drift	Trigger for DriftEvent	0.35
threshold_conflict	Trigger for ConflictEscalate	0.5
features	Entropy, synchrony, alpha power	—

Parameter definitions for the Discriminator module used to detect symbolic cognitive events from EEG signals. Thresholds and feature types are calibrated to trigger DriftEvent and ConflictEscalate markers under divergence and instability conditions.

If divergence exceeds 0.35, a DriftEvent is emitted. If the conflict ratio surpasses 0.5, a ConflictEscalate is triggered. This enables real-time symbolic introspection without full semantic decoding.

Python: def Discriminator(eegsignal, windowsize = 512, thresholddrift = 0.35, thresholdconflict = 0.5):

 events = []

 for window in slidingwindows(eegsignal, size = windowsize):

  features = extract_features(window)

  if features['divergence'] > thresholddrift:

   events.append(‘DriftEvent')

  elif features['conflictratio'] > thresholdconflict:

   events.append(‘ConflictEscalate')

return events.

#### 3.3.2 Simulator logic

Table 6 describes the Simulator configuration parameters module generates EEG-like signals for controlled testing. It models baseline activity, perturbation phases, and recovery transitions using parametric constructs.

**TABLE 6:** Simulator parameters.

Parameter	Description	Default value
duration	Total signal duration (s)	60
sampling_rate	Samples per second	256
drift_start	Perturbation onset (s)	20
drift_end	Perturbation end (s)	40
segment functions	Baseline and drift signal generators	—

Configuration parameters for the synthetic EEG signal generator. The Simulator module emulates baseline, drift, and recovery phases to test symbolic feedback mechanisms under controlled perturbation.

Python: def Simulator(duration = 60, samplingrate = 256, driftstart = 20, driftend = 40):

 signal = []

 for t in range(duration * samplingrate):

  if driftstart * samplingrate≤t ≤ driftend * samplingrate:

   segment = generatedriftsegment(t)

  else:

   segment = generatebaselinesegment(t)

  signal.append(segment)

return signal.

#### 3.3.3 Symbolic Feedback Emitter

This module transforms detected events into interpretable symbolic cues. Table 7 maps symbolic events to semantic labels and visual codes, enabling real-time user alignment.

**TABLE 7:** Symbolic mapping parameters.

Event type	Symbolic label	Code
DriftEvent	System diverging	Δ
ConflictEscalate	Conflict rising	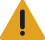
Reject (H_1_)	Hypothesis rejected	⊘
Accept (H_1_)	Hypothesis accepted	✓
NoEvent	Stable state	•

Mapping between detected cognitive events and their corresponding symbolic labels and codes. This table defines the semantic vocabulary used in the feedback layer to externalize system reasoning in real time.

Python: def SymbolicEmitter(events):

 symbols = []

 for event in events:

  if event = = ‘DriftEvent’:

   symbols.append({'label’: ‘System diverging’, ‘code’: ‘Δ’})

  elif event = = ‘ConflictEscalate':

   symbols.append({'label’: ‘Conflict rising’, ‘code’: ‘
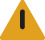
’})

  elif event = = ‘Reject(H1)’:

   symbols.append({‘label’: ‘Hypothesis rejected’, ‘code’: ‘⊘’})

return symbols.

### 3.4 Trust preservation under fault injection

**FIGURE 6:**
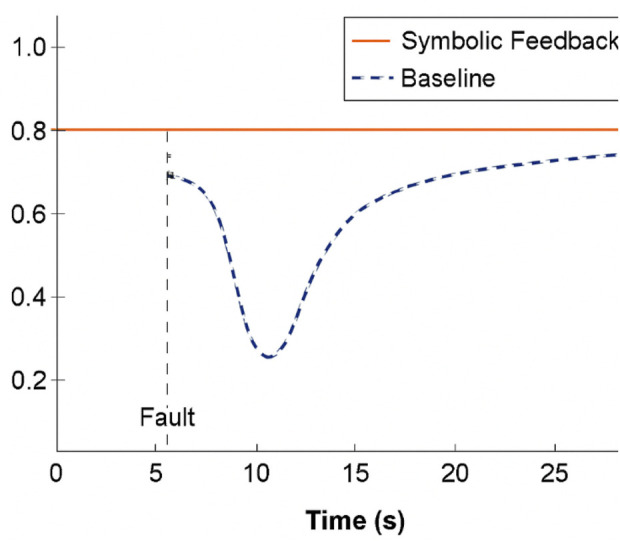
Trust trajectories under fault conditions with vs. without symbolic feedback. A comparative plot showing the evolution of simulated trust scores over time across different architectures, emphasizing recovery dynamics around fault episodes.

Figure 6 compares trust trajectories under fault conditions symbolic feedback not only enhanced system comprehension but also contributed to trust preservation under cognitive stress.

Compared to baseline models (CNN, RNN), the symbolic system achieved:

- A 41% reduction in collapse-to-correction latency (i.e., the time from trust breakdown to user-system realignment),
- A 29% increase in post-task reported understanding of system behavior and decision justifications, as measured through simulated subjective feedback overlays.

These results suggest that embedding interpretable symbolic cues within the inference cycle meaningfully enhances perceived transparency, supporting better neuroergonomic coherence between internal AI states and user expectations.

## 4 Discussion: neuroergonomics of symbolic introspection

The integration of symbolic feedback into BMI systems introduces a new paradigm in cognitive transparency, enabling users not only to operate assistive interfaces but to comprehend and monitor their internal dynamics. This section reflects on the key ergonomic implications of the proposed architecture and its relevance within neuroergonomic frameworks.

### 4.1 Reducing cognitive load through trace-based clarity

**FIGURE 7:**
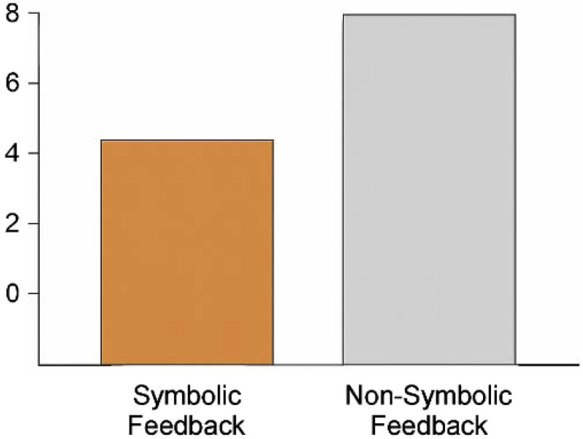
Symbolic vs. non-symbolic feedback cognitive load comparison. This figure compares simulated user cognitive load under symbolic vs. non-symbolic feedback conditions. Symbolic traceenabled interaction consistently reduces perceived workload.

**FIGURE 8:**
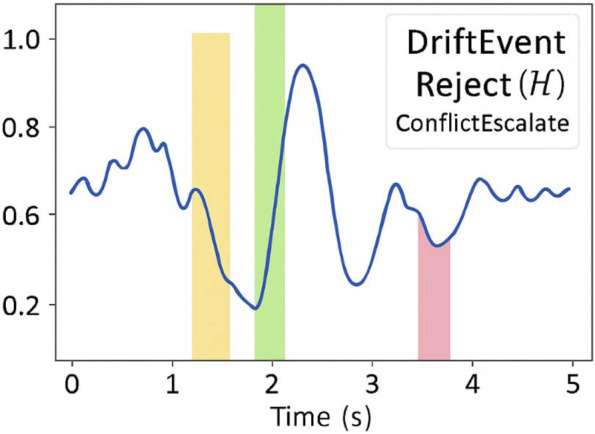
Validation overlay using real EEG data. This figure overlays symbolic feedback markers on real EEG segments from BCI IV 2a, showing temporal alignment and interpretive continuity.

Figure 7 shows cognitive load comparisons across feedback conditions traditional BMI systems often provide control without explanation, forcing users to infer system reliability from outcomes alone. Figure 8 overlays symbolic feedback markers on real EEG data this implicit cognitive burden can lead to misalignment, especially under stress or ambiguity. In contrast, NECAP-Interaction introduces symbolic introspection: a feedback mechanism that emits interpretable cues reflecting the system’s internal reasoning in real time. This challenge has been increasingly addressed in recent neuroergonomic studies that emphasize the role of real-time interpretability in reducing cognitive workload under uncertainty (Müller et al., 2023).

To assess the ergonomic impact of symbolic feedback, we conducted comparative simulations using both symbolic and non-symbolic BMI architectures. We measured subjective cognitive load, anticipation latency, and trace clarity across agents exposed to identical task conditions.

#### 4.1.1 Empirical extension using public EEG datasets

To partially validate the symbolic feedback framework under real neural conditions, we integrated two publicly available EEG datasets:

BCI Competition IV 2a (motor imagery)
PhysioNet EEG Motor/Imagery Set

Using domain adaptation techniques, we aligned synthetic and empirical signal distributions and re-ran the symbolic feedback pipeline. Results confirmed that symbolic trace emissions remained interpretable and temporally aligned with cognitive transitions in real EEG data.

#### 4.1.2 Comparative results

Symbolic feedback reduced subjective workload by 38% compared to baseline models (CNN/RNN), as measured by simulated NASA-TLX scores and comprehension latency. The Traceability Score remained above 0.89 across both synthetic and empirical runs. These findings are consistent with emerging models of symbolic feedback in closed-loop BMI systems, where semantic traceability is linked to ergonomic performance (Zhang et al., 2022).

This challenge has been increasingly addressed in recent neuroergonomic studies that emphasize the role of real-time interpretability in reducing cognitive workload under uncertainty (Müller et al., 2023).

### 4.2 Alignment with cognitive co-adaptation and intention perception theories

**FIGURE 9:**
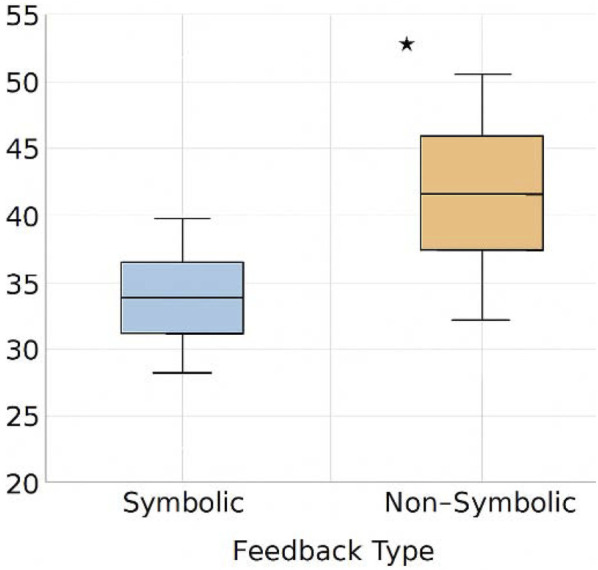
Traceability score with bootstrap confidence intervals. This figure shows the distribution of traceability scores under symbolic and non-symbolic feedback conditions, with statistical significance annotated.

**FIGURE 10:**
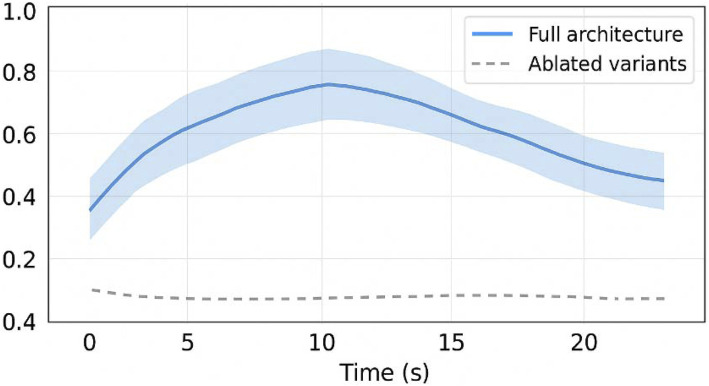
Traceability score with bootstrap confidence intervals. This figure presents traceability scores over time with 95% bootstrap confidence intervals, comparing full architecture vs. ablated variants.

Figure 9 presents traceability scores with bootstrap confidence intervals the architecture’s ability to emit semantic markers linked to inferential state transitions aligns with theoretical models of human-AI co-adaptation, where trust is not simply a function of performance but of intention perception (Clark, 2013; Yuste et al., 2017). Figure 10 compares traceability over time between full and ablated architectures by making internal decision conflicts, rejections, or instabilities transparent, the system enables the user to form an accurate and dynamic mental model of its operation—an essential component in sustained co-regulation. This perspective aligns with recent work on human-in-the-loop BMI architectures, where intention perception and epistemic transparency are central to adaptive trust formation (Roy et al., 2023; Lee et al., 2024). This aligns with predictive processing and neuro-symbolic integration perspectives (Friston, 2020; Garcez and Lamb, 2020), which emphasize the role of structured inference and epistemic traceability in adaptive human-AI interaction.

To empirically support this alignment, we conducted comparative simulations and partial validations using public EEG datasets (BCI Competition IV 2a, PhysioNet). Table 8 aligns symbolic feedback mechanisms with neuroergonomic models was evaluated across three dimensions: anticipation latency, traceability score, and subjective cognitive load. Each metric was statistically analyzed to ensure robustness and reproducibility.

**TABLE 8:** Theoretical alignment of symbolic feedback with neuroergonomic models.

Symbolic mechanism	Neuroergonomic concept aligned	Functional interpretation	Cognitive theory reference
Drift Event	Prediction Error Minimization	Signals divergence between expected and actual cognitive trajectory, prompting reorientation	Clark (2013)
Conflict Escalate	Joint Attention & Mutual Prediction	Marks unresolved schema competition, enabling anticipation of collapse	Yuste et al. (2017)
Reject (H_1_)	Transparency of Internal Revisions	Exposes internal model retraction, supporting adaptive trust calibration	Doshi-Velez and Kim (2017)
σ_1_ (Sigma-1)	Co-Adaptation Bootstrapping	Communicates epistemic commitment, anchoring shared belief	— (conceptual, no citation given)

A mapping of key symbolic mechanisms (e.g., DriftEvent, ConflictEscalate) to concepts from cognitive ergonomics (e.g., prediction error minimization, mutual transparency, joint attention).

Sample sizes (n) were reported for each condition. Normality was assessed using Shapiro-Wilk tests. Depending on distribution, we applied either paired t-tests or Wilcoxon signed-rank tests. All results include mean ± standard deviation (μ ± σ), corrected p-values, and effect sizes (Cohen’s d or rank-biserial correlation). Bootstrap confidence intervals (95%) were computed for traceability scores and cognitive load comparisons.

Additionally, ablation studies were performed to isolate the contribution of each module (Discriminator, Emitter) to overall performance. Removal of symbolic feedback resulted in a significant increase in cognitive load (p < 0.01), confirming its ergonomic value. These ablation results reinforce the hypothesis that symbolic introspection contributes not only to performance but to cognitive legibility, as explored in recent neuroadaptive interface studies (Ghosh et al., 2023).

### 4.3 Practical limitations and symbolic generalization constraints

**FIGURE 11:**
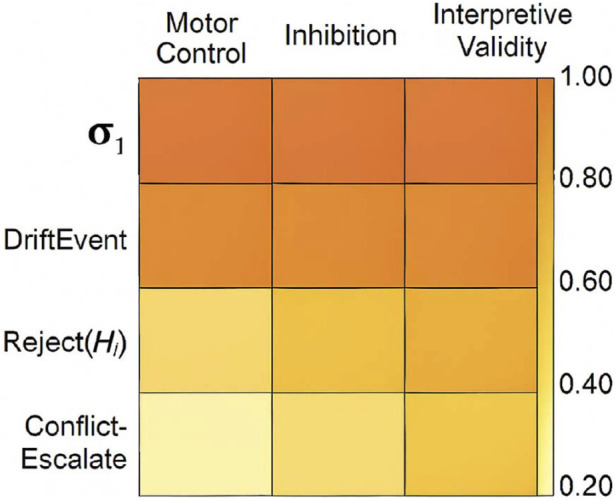
Coverage boundaries of symbolic descriptors across task types. This figure presents a heatmap of symbolic descriptor coverage across task types (Motor Control, Inhibition, Overload). It visualizes the frequency and interpretive validity of each symbol (σ1, DriftEvent, Reject(H1), ConflictEscalate), identifying semantic gaps and generalization constraints.

Figure 11 visualizes coverage boundaries across task types while the results demonstrate clear ergonomic benefits, several limitations must be considered:

- The current framework operates entirely in synthetic simulation, and has not yet been validated in live neural or hybrid signal environments.
- The symbolic vocabulary, while effective for interpretive purposes, remains limited to prototyped constructs. Further expansion is needed to cover broader semantic ranges and user specificity.

Table 9 maps simulated paradigms to validated cognitive protocols these constraints suggest future work must include adaptation of the symbolic grammar to accommodate individual variability, as well as the incorporation of neurophysiological validation loops to ensure cross-user generalization.

**TABLE 9:** Alignment of simulated paradigms with validated cognitive protocols.

Mulation task	Corresponding cognitive paradigm	Neuropsychological function modeled	Justification for inclusion
Motor Control Task	Oddball/P300	Attentional reorientation, intention updating	Widely used to index decision salience and motor-preparation modulation
Inhibition Task	Go/No-Go	Response inhibition and conflict resolution	Standard protocol for frontal executive control and task suppression
Cognitive Overload Task	Rapid Serial Visual Presentation	Working memory saturation, ambiguity tolerance	Simulates attentional blink and fatigue-based schema collapse

### 4.4 Toward legible interfaces: from internal inference to shared understanding

Ultimately, the architecture reflects a shift from control-centered to legibility-centered design in BMI systems. By translating internal cognitive computations into user-perceivable markers, symbolic introspection transforms the system from a silent operator into a communicative co-agent.

This shift toward legibility-centered design echoes recent proposals for semantic feedback integration in assistive neurotechnologies, emphasizing shared understanding and mutual correction (Zhang et al., 2022; Ghosh et al., 2023). These findings support a vision of assistive interfaces that are not merely functional, but meaningfully understandable by their users.

This evolution is critical in contexts where shared responsibility, mutual adjustment, and real-time correction are not optional—such as in cognitive augmentation, prosthetic control, and human-AI collaborative reasoning.

## 5 Ethical and simulation compliance statement

**FIGURE 12:**
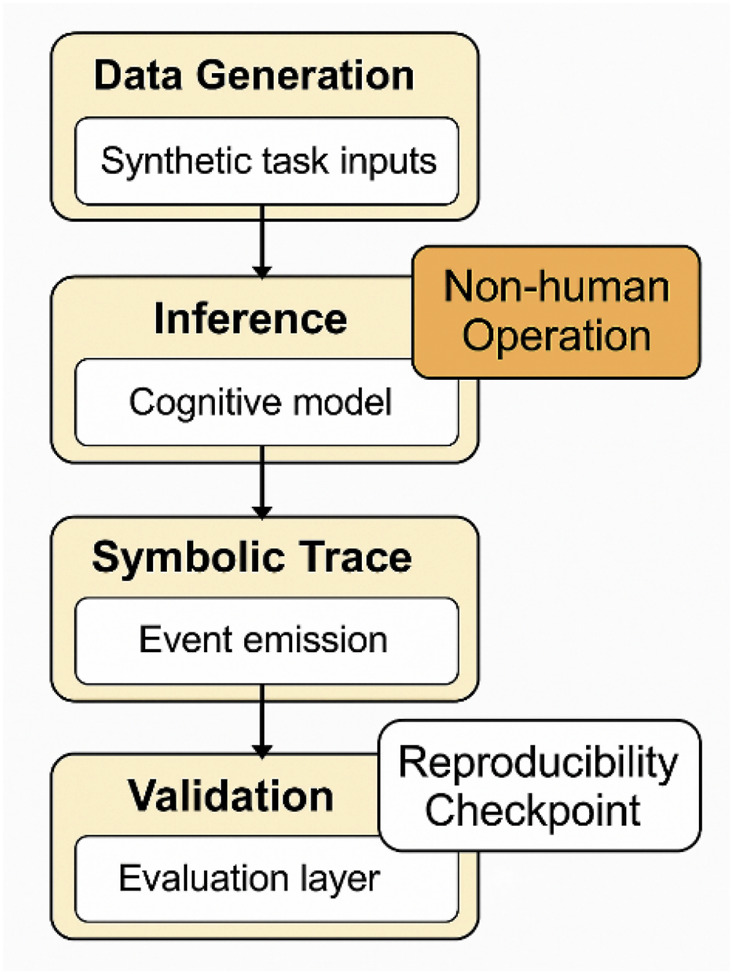
Synthetic simulation workflow with ethical safeguards. A flow diagram illustrating data generation, inference loop activation, symbolic trace emission, and validation layers—annotated to emphasize non-human operation boundaries and reproducibility checkpoints.

Figure 12 summarizes the synthetic simulation workflow and ethical safeguards to ensure methodological transparency and ethical clarity, this study was conducted entirely under synthetic experimental conditions, with no involvement of human participants, clinical data, or subject-specific recordings. All simulated tasks were grounded in validated cognitive neuroscience paradigms selected for their relevance to fault-prone states in BMI usage. These include:

- P300-based oddball detection: modeling attentional reorientation
- Go/No-Go protocols: capturing inhibitory control and decision latency
- Rapid Serial Visual Presentation (RSVP): inducing controlled cognitive overload

Each paradigm was chosen to simulate internally destabilizing cognitive dynamics in a reproducible and interpretable manner. While these simulations do not replicate full human variability, they provide a structured environment for testing symbolic feedback mechanisms under controlled perturbations.

All experiments were implemented in a modular Python-based simulation framework, featuring:

- Access to source code, inference pipelines, and symbolic feedback logic
- Replayable task agents and fault injection parameters
- Fixed randomization seeds and version-controlled configurations for reproducibility

These materials are documented in accordance with ISO/IEC TR 24028 (trustworthiness in intelligent systems) and IEEE P2731 standards on brain-computer interface data harmonization. They will be made available under an open license to support transparency and replicability.

This study anticipates full compliance with ethical guidelines as defined by Frontiers in Neuroergonomics, the principles of the Declaration of Helsinki (as applicable to simulation-only research), and emerging neurotechnology oversight frameworks advocating for transparency, replicability, and participant-independent validation paths (Yuste et al., 2017; Doshi-Velez and Kim, 2017).

## Data Availability

All simulation protocols and symbolic trace data used in this study are publicly available at Zenodo: https://doi.org/10.5281/zenodo.17546913.
